# The use of point of care gastric ultrasound and anesthesia management in pediatric patients with preoperative fasting non-adherence scheduled for elective surgical procedures: a retrospective study

**DOI:** 10.1186/s12871-024-02628-0

**Published:** 2024-07-15

**Authors:** Marc D. Mecoli, Kirti Sahu, Joseph W. McSoley, Lori A. Aronson, Suryakumar Narayanasamy

**Affiliations:** 1 Department of Anesthesia, Cincinnati Children’s Hospital Medical Center, Cincinnati, OH USA; 2 University of Cincinnati College of Medicine, Cincinnati, OH USA; 3 Department of Anesthesiology, Cincinnati Children’s Hospital Medical Center, 3333 Burnet Avenue MLC 2001, Cincinnati, OH 45229 USA

**Keywords:** Gastric ultrasound, Point of care testing

## Abstract

**Background:**

Failure to adhere to perioperative fasting requirements increases aspiration risk and can lead to delay or cancellation of surgery. Point of care gastric ultrasound may guide decision-making to delay, cancel or proceed with surgery.

**Methods:**

This study aimed to describe gastric contents using point of care gastric ultrasound in pediatric patients with known fasting guideline violations presenting for elective surgery. This was a single-center retrospectivechart review of gastric ultrasound scans in patients presenting for elective surgeries with “nothing by mouth” violation (per fasting guidelines) or unclear fasting status. The primary outcome is description of gastric contents using point of care ultrasound. The ultrasound findings were classified as low-risk for aspiration (empty, clear fluid < 1.5 ml/kg), high-risk (solids, clear fluid > 1.5 ml/kg), or inconclusive study. Gastric ultrasound findings were communicated to the attending anesthesiologist. For patients proceeding without delay the estimated time saved was defined as the difference between ultrasound scan time and presumed case start time based on American Society of Anesthesiologists fasting guidelines.

**Results:**

We identified 106 patients with a median age of 4.8 years. There were 31 patients (29.2%) that had ultrasound finding of high-risk gastric contents. These patients had cases that were delayed, cancelled or proceeded with rapid sequence intubation. Sixty-six patients (62.3%) were determined to be low-risk gastric contents and proceeded with surgery without delay. For these patients, a median of 2.6 h was saved. No aspiration events were recorded for any patients.

**Conclusions:**

It is feasible to use preoperative point of care gastric ultrasound to determine stomach contents and risk-stratify pediatric patients presenting for elective surgical procedures with fasting non-adherence. Preoperative gastric ultrasound may have a role in determining changes in anesthetic management in this patient population.

## Introduction

Pulmonary aspiration is a dreaded anesthetic complication that contributes to significant postoperative morbidity and mortality [1, 2]. Despite many technological advances in perioperative care, pulmonary aspiration is continuing to be the leading cause of death in airway management related complications [3]. Perioperative fasting guidelines aim to reduce the risk of pulmonary aspiration by ensuring an empty stomach at the time of anesthetic induction in healthy patients [4, 5]. However, these guidelines do not consider individual risk factors affecting gastric motility and can vary in different societies and institutions [5–7]. Nothing by mouth (NPO) guideline violations, defined as not following appropriate fasting instructions, have a reported incidence of 1.5 − 4.5% in the pediatric population. Causes of NPO violations include lack of understanding of NPO instructions, patient eating without parental knowledge, scheduling changes in surgical case time, language barriers, and an inconsolable child, among many others [8, 9]. Regardless of the reason, case cancellations can lead to delays in access to care, inconvenience and frustration for the patients and their families [10]. 

Point of care (POC) gastric ultrasound provides objective, real-time assessment of gastric content and volume. It has been shown to be a reliable diagnostic modality for accurately assessing gastric content and volume [11–19]. POC gastric ultrasound assessment of gastric contents could reduce the risk of pulmonary aspiration [20]. Gastric ultrasound evaluation may minimize case delays, reduce cancellations, and allow for safer anesthetic management by objectively assessing gastric contents in patients at increased risk for aspiration, including patients with NPO violations [14–16, 21]. The exact amount of gastric volume that is at risk for aspiration or safe is still unknown and debated [22]. Although the safest minimum gastric volume is unknown, it has been generally accepted that clear liquids less than 1.5 ml/kg is consistent with baseline gastric secretions and even volumes higher than 1.5 ml/kg could be present in 1-9% of appropriately fasted patients depending on the study population [13, 21, 23]. Gastric volume greater than 1.5 ml/kg and the presence of solid contents can increase the risk of aspiration related complications. [17, 18]. We have been using gastric ultrasound to objectively verify gastric contents in pediatric patients for the past few years. The purpose of this retrospective study was to describe the POC gastric ultrasound findings and anesthetic management in pediatric patients with known NPO violations presenting for surgery.

## Methods

### Study design and population

Institutional Review Board (IRB) approval was obtained prior to study commencement (IRB# 2023 − 0292). Existing records of all gastric ultrasound scans performed in perioperative patients presenting for *elective* surgical procedures with known NPO violation based on ASA preoperative fasting guidelines [4] or unclear NPO status between December 2022 and April 2023 were included.

The primary outcome was the preoperative ultrasound findings of the gastric contents. The ultrasound findings were classified as low-risk for aspiration (empty, clear fluid < 1.5 ml/kg), high-risk (solids, clear fluid > 1.5 ml/kg), or inconclusive study as previously described by Spencer and colleagues [23]. Secondary outcomes include the estimated time saved in patients with gastric ultrasound as compared to following standard ASA fasting guidelines.

### Gastric ultrasound

Per institutional practice, gastric ultrasound scans are performed by a small subset of anesthesiologists experienced in point-of-care (POC) gastric ultrasound exams at the request of the anesthesia team caring for the patient. The anesthesiologists performing gastric ultrasound at our institutional have all previously participated in the departmental POC gastric ultrasound education curriculum using the I-AIM framework described in the 2021 American Society of Regional Anesthesia and Pain Medicine expert panel recommendations on point-of-care ultrasound education and training [12, 24]. 

All gastric ultrasound scans are performed using a 3-5 MHz curvilinear probe (Venue G.O. or LOGIQ S7 [G.E. Healthcare, Chicago, IL, USA] or TE7 [Mindray, Mahwah, NJ, USA] ultrasound machines). All patients are scanned in the right lateral decubitus position with the probe in a parasagittal orientation. The gastric antrum is visualized at the aorta and superior mesenteric artery level. The predicted antral volume for clear fluids is determined using a cross-sectional area as described by Spencer and colleagues [23]. A procedure note describing ultrasound findings and interpretation is placed in the medical record and communicated to the family and the anesthesiologist assigned to the case. The anesthesia team determines case management, induction technique, and choice of airway.

For the study, the following data were extracted and recorded on a data sheet: age, gender, surgical procedure, NPO times, gastric ultrasound findings, induction technique, case delay or cancellation, airway used, and aspiration events. For patients proceeding without delay, the estimated time saved was calculated as the difference between ultrasound scan time and presumed case start time based on ASA fasting guidelines (2 h for clears, 6 h for milk/formula, 8 h for solids). Cost savings analysis was performed using customary operating room service charges published by Cincinnati Children’s Hospital Medical Center under Sect. 3727.42 of Ohio Revised Code. OR charge for first 15 min = $3,285, and additional 15 min = $1,034.

### Study site

Cincinnati Children’s Hospital Medical Center (CCHMC) is a 673-bed non-profit organization serving as the University of Cincinnati Academic Health Center’s major teaching facility for pediatrics and the only children’s hospital in the Cincinnati metropolitan area (population of 2.3 million). CCHMC performs more than 45,000 anesthetics annually.

### Statistical analysis

Statistical analysis was performed using Microsoft Excel software. Descriptive data, median and interquartile ranges (IQR) for continuous variables, and frequencies and percentages for categorical variables were analyzed.

## Results

During the 15-month study period, we identified 106 patients with gastric ultrasound examinations performed for NPO violation prior to non-urgent surgical procedures. Eight anesthesiologists certified in POC gastric ultrasound performed gastric ultrasound examinations in the study cohort. Demographic data are presented in Table 1.

**Table 1:** Demographic characteristics

Age (median years, IQR)	4.8 (2.5, 9.4)
**Sex** (frequency, %)	
Male Female	57 (53.8%) 49 (46.2%)
**ASA Physical Status** (frequency, %)	
I II III Missing	24 (22.6%) 56 (52.8%) 21 (19.8%) 5 (4.7%)
**Surgery Type** (frequency, %)	
ENT Dental Urology General Surgery Gastroenterology Other	27 (25.4%) 23 (21.7%) 21 (19.8%) 10 (9.4%) 7 (6.6%) 18 (17%)

ASA: American Society of Anesthesiologists, ENT: Ear, Nose, and Throat,

IQR: interquartile range

*Low-risk*: Ultrasound evidence of gastric antrum consistent with low risk for aspiration (defined as an empty gastric antrum or clear fluids < 1.5 ml/kg) was noted in 66 patients (62.3%) identified as NPO violators as per the current ASA guidelines. All of these patients proceeded with surgery without delay. A median of 2.6 h (IQR 1 to 3.5) was saved for these patients. This represents median $10,523 (IQR $6,387 to $16,727) operating room cost as estimated by customary per hour operating room service charges. *High-risk*: Ultrasound evidence of high-risk antrum (clear liquids > 1.5 ml/kg or presence of any amount of solids) was noted in 31 *(29.2%) patients.* Only two patients had clear fluid greater than 1.5 ml/kg. One surgery proceeded after a two-hour wait. The other patient was asked to wait, but the family rescheduled the case. In patients with thick or solid material (*n* = 29), 22 (76%) cases were canceled. Five cases were delayed to comply with the appropriate NPO guidelines and proceeded as usual. Three cases were delayed to comply with NPO guidelines and proceeded with rapid sequence induction (RSI). One patient’s procedure was deemed urgent by the surgeon, secondary to a dental abscess, and the anesthesia team proceeded with RSI after five hours of fasting after the patient ate low-fat yogurt.

Gastric ultrasound findings, risk level assessment, and management decision data are summarized in Table 2. No aspiration events were recorded for any of the patients.

**Table 2:** Gastric ultrasound findings, risk level, and management decision (*n* = 106) for patients with NPO violation

Gastric Ultrasound Findings Frequency (%)		Risk Level Frequency (%)		Management Decision Frequency (%)	
Empty gastric antrum	38 (35.8%)	Low	66 (62.3%)	Proceed without delay	66 (62.3%)
Clear liquids < 1.5mL/kg	28 (26.4%)				
Clear liquids > 1.5mL/kg	2 (1.9%)	High	31 (29.2%)	Case canceled	22 (20.8%)
Thick material or solids	29 (27.4%)			Case delayed	8 (7.5%)
				Proceed with RSI	1 (0.9%)
Inconclusive	9 (8.5%)	NA		NA	

RSI = rapid sequence intubation, NA = not applicable

## Discussion

Our study demonstrated that gastric ultrasound provided objective evidence which may have guided decision-making for patients with known NPO violations or unclear NPO status in pediatric patients undergoing elective surgery. Approximately 60% of the patients had ultrasound evidence of either an empty antrum or low-volume clear fluid, and the elective surgeries proceeded without delay. This positively impacts the family experience and improves operating room utilization.

The gastric fluid volume that minimizes the risk of pulmonary aspiration is still being debated [22]. Multiple ultrasound and endoscopic studies demonstrated that up to 95–97% of appropriately fasted children have a residual gastric volume < 1.25–1.5 ml/kg [23, 25]. We used 1.5 ml/kg as the threshold to minimize the risk of unnecessary cancellation because children are rarely anesthetized immediately following gastric scanning. The intake nurse confirms NPO verification within the first few minutes of assessment in Same-Day Surgery. Confirmation triggers a call for POC gastric ultrasound, where gastric ultrasound is performed well before induction time in the preoperative holding area. While we do not have formal documentation of the scan time duration, the scan typically takes less than five minutes during the preoperative evaluation stage. POC gastric ultrasound does not cause a delay to the start of the procedure. We believe most patients at or close to the 1.5 ml/kg threshold reached a lower volume by anesthesia induction. In patients with inconclusive ultrasound scans due to patient movement, colonic air artifact or other factors, the decision to proceed, delay or cancel these procedures was made without information from the ultrasound study by the primary anesthesiologist provider. In patients with solid material in the stomach, the decision to cancel, delay or perform RSI was at the attending anesthesiologist’s discretion.

Presumably, many of the cases with known NPO violations would have been canceled, although our study could not determine that outcome. Reducing case cancellation and delays positively impacts patient care, family satisfaction, and improves operating room utilization. For the cases proceeding without delay we estimated saving a median 2.6 h of operating room time per patient. We estimate up to $10,523 in lost operating room charges could have been saved per patient. However, this does not take into account being able to adjust operating room scheduled to move up other patients when delay occurs because of NPO violation. Prospective studies and more detailed cost-savings analysis may be warranted to better understand the effect of gastric ultrasound on operating room utilization.

Our findings are similar to previously published reports in adult patients. Alakkad et al. showed that preoperative gastric ultrasound in adult patients with known NPO non-adherence undergoing elective surgical procedures led to changes in anesthetic management in 71% of the patients [16]. Approximately half of the patients had a revised surgery time with a trend toward a lower incidence of surgical delays. Similarly, Van de Putte et al. used gastric ultrasound in 37 adult patients with NPO violations. They found that gastric ultrasound helped change anesthetic management in 54% of cases, with a trend toward lower case cancellations and delays [14]. 

Serial gastric ultrasound examination has been used to demonstrate decreasing gastric contents over time in patients being considered for procedural sedation in the emergency department [26]. Gagey et al. demonstrated qualitative gastric ultrasounds may change anesthetic management in pyloromyotomy patients, as 88.2% no longer required RSI after aspiration of stomach contents [27]. 

In our study, we identified some patients with a large amount of clear liquid (> 1.5 ml/kg) in whom gastric ultrasound was used to serially monitor the gastric content over time to ensure it was below the threshold before proceeding with anesthesia.

The present study has several limitations. Due to the study’s retrospective nature, we could not control the decision to perform gastric ultrasound in patients with NPO violations. We did not always have access to detailed information about NPO status, only that there was a known violation or unclear NPO status. We were not able to determine the degree to which the ultrasound findings influenced the anesthesiologist’s decisions to proceed with, delay or cancel cases. This was a single-center study with multiple anesthesiologists with expertise in performing gastric ultrasound quickly. The generalizability of the results to other institutions needs to be confirmed with future prospective multicenter trials. Finally, although no patients in the study were noted to have pulmonary aspiration, our sample size is too small to determine the overall influence of gastric ultrasound on patient safety outcomes due to the low incidence of pulmonary aspiration in pediatric surgical patients.

Using POC gastric ultrasound as a risk assessment tool is feasible to determine gastric contents in pediatric surgical patients with known NPO violations and may be helpful in formulating an appropriate anesthetic plan. Further studies are needed to determine the type and timing of oral intake that warrants performing gastric ultrasound in pediatric patients with known NPO violations.

## Conclusion

We studied the use of preoperative gastric ultrasound in preoperative patients scheduled for elective surgical procedures who had suspected or known NPO violations. We demonstrated that risk stratification based on gastric ultrasound findings is feasible and may associated with differences in anesthesia decision-making.

## Data Availability

Availability of data and materials: The datasets used and/or analyzed during the current study are available from the corresponding author upon reasonable request.

## Abbreviations

ASA: American Society of Anesthesiologists
CCHMC: Cincinnati Children’s Hospital Medical Center
IRB: Institutional Review Board
NPO: Nil per os / Nothing by mouth
POC: Point of care
RSI: Rapid sequence induction
STROBE: Strengthening the reporting of observational studies in epidemiology
